# Distinct Spatio-Temporal Dynamics of Tumor-Associated Neutrophils in Small Tumor Lesions

**DOI:** 10.3389/fimmu.2019.01419

**Published:** 2019-06-25

**Authors:** Simon Sody, Mohib Uddin, Anika Grüneboom, André Görgens, Bernd Giebel, Matthias Gunzer, Sven Brandau

**Affiliations:** ^1^Department of Otorhinolaryngology, University Hospital Essen, University Duisburg-Essen, Essen, Germany; ^2^Respiratory Global Medicines Development (GMD), AstraZeneca, Gothenburg, Sweden; ^3^Institute for Experimental Immunology and Imaging, University Hospital Essen, University Duisburg-Essen, Essen, Germany; ^4^Institute for Transfusion Medicine, University Hospital Essen, University Duisburg-Essen, Essen, Germany; ^5^Department of Laboratory Medicine, Karolinska Institutet, Stockholm, Sweden

**Keywords:** tumor-associated neutrophils, neutrophil granulocytes, intravital imaging, multiphoton microscopy, tumor microenvironment, tumor immunology, CXCR2, AZD5069

## Abstract

Across a majority of cancer types tumor-associated neutrophils (TAN) are linked with poor prognosis. However, the underlying mechanisms, especially the intratumoral behavior of TAN, are largely unknown. Using intravital multiphoton imaging on a mouse model with neutrophil-specific fluorescence, we measured the migration of TAN in distinct compartments of solid tumor cell lesions *in vivo*. By longitudinally quantifying the infiltration and persistence of TAN into growing tumors in the same animals, we observed cells that either populated the peripheral stromal zone of the tumor (peritumoral TAN) or infiltrated into the tumor core (intratumoral TAN). Intratumoral TAN showed prolonged tumor-associated persistence and reduced motility compared to peritumoral TAN, whose velocity increased with tumor progression. Selective pharmacological blockade of CXCR2 receptors using AZD5069 profoundly inhibited recruitment of TAN into peritumoral regions, while intratumoral infiltration was only transiently attenuated and rebounded at later time points. Our findings unravel distinct spatial dynamics of TAN that are partially and differentially regulated via the CXCR2 signaling pathway.

## Introduction

During the last decade, a high intratumoral frequency of tumor-associated neutrophils (TAN) was established as a strong predictor of poor clinical outcome in the majority of solid tumor entities (1–3). In fact, in a recent transcriptomic analysis of ~18.000 human tumor samples from 14 solid tumors neutrophils (secondary to mast cells) showed the strongest correlation with adverse cancer outcomes (1).

Despite this well-established prognostic role in the clinical setting, the mechanisms underlying a disease-promoting activity of neutrophils are still poorly understood. Murine studies have identified a variety of neutrophil-mediated pro-tumorigenic factors (4). Production of neutrophil-derived matrix metalloproteinases like MMP-9 were shown to release VEGF-A from the extracellular matrix (ECM) and thereby constitute a major source of pro-angiogenic factors in the tumor microenvironment (5–8). In addition, neutrophils can influence invasiveness and metastatic potential of tumor cells by angiotropism (9) or neutrophil granule-derived enzymes, that actively remodel the ECM and mutually activate tumoral proteases to promote the invasion of tumor cells (10). Priming of lung pre-metastatic niches through neutrophil MMPs enhanced the metastatic spread of mammary tumors (11). Likewise, human neutrophils, after CXCR2-dependent recruitment (12), and MAPK activation have the ability to induce multiple tumor promoting mechanisms (6), which includes the cortactin-mediated induction of tumor cell invasion and metastasis in patients (13). Further, immunosuppressive neutrophils with myeloid-derived suppressor cell activity (PMN-MDSC) can drive tumor immune evasion (14–16).

Despite this important role of neutrophils in malignant disease, until recently, even in murine models, mechanistic studies on the recruitment and intratumoral biology of TAN were limited to histological tissue analyses, *ex vivo* investigations or depletion of neutrophils by antibodies without a direct observation of the live cells within the tumor. To a large extent, this has been based on existing models such as lys-EGFP, c-fms-EGFP, and hMRP8-Cre that were not neutrophil specific and hence also included the analysis of “contaminating” cells from the myelomonocytic and dendritic lineages (17–19). As such, immune-mediated mechanisms of neutrophil recruitment to the sites of tumor are incompletely understood.

Experimental murine studies and clinical correlation analyses have identified ligands for CXCR2 as major drivers of TAN recruitment into tumor lesions, involving CXCL1/KC, CXCL2/MIP-2, CXCL5/LIX, CXCL6, and MIF (12, 20–23). Consequently, at least in murine models, many of the disease-promoting effects of neutrophils can be attenuated by CXCR2 blockade (24–26). In contrast to human neutrophils, where CXCR1 and CXCR2/IL-8 interaction is a major chemoattractant (27), in mice, CXCR1 has a redundant capacity for neutrophil trafficking whilst playing a predominant role in regulating degranulation (28). Neutrophil effector functions and trafficking to tissues are also context-dependent. While neutrophils were initially considered as purely pathogen-clearing innate effector cells, to date, complex and adaptable functions in infection, inflammation and cancer are emerging (29, 30).

In this study, we used AZD5069 to modulate recruitment of TAN into tumor lesions *in vivo*. AZD5069 is a small molecule antagonist with over 100-fold selectivity for CXCR2 relative to CXCR1 receptor, that does not adversely affect neutrophil-mediated host immunity (31, 32). Beyond a potential immuno-oncological target (20), AZD5069 has been extensively studied as an orally active immunotherapy in chronic respiratory diseases, including COPD (33, 34), bronchiectasis (35) and severe asthma (36, 37). In murine tumor models CXCR2 blockade has been shown to modulate neutrophil trafficking to sites of chronic inflammation, subsequently reduced tumor and metastasis formation and enhanced treatment efficacy in distinct therapeutic conditions (38–41).

To directly image different phases of neutrophil invasion into locally growing tumors, we used a recently established mouse model, termed Catchup (42). This allowed us to uncover time-dependent changes in frequency, localization, and migratory patterns of neutrophils in small tumor lesions. We found that neutrophils localized in either intratumoral or peritumoral regions revealed distinct migratory patterns. Surprisingly, blockade of the CXCR2 chemokine receptors, previously believed to selectively inhibit migration of neutrophils into tumors and other inflammatory tissue lesions, was shown to markedly attenuate peritumoral stromal TAN, whilst only transiently blocking the recruitment of TAN into the early tumor cell lesion. These findings have important implications for the precision targeting of TAN in emerging cancer combination immunotherapies.

## Materials and Methods

### Animals

All animal experiments were performed in accordance with the animal ethics committee of the state of North Rhine–Westphalia, Germany, and German guidelines for experimental animal welfare. Generation of Catchup^IVM−red^ was previously described (42). Catchup^IVM−red^ mice were bred in the animal facility of the Centre for Medical Biotechnology of the University Duisburg Essen and housed under specific pathogen-free conditions in individually ventilated cage racks. Both male and female Catchup^IVM−red^ mice were used at any age between 3 and 6 month, but animals were sex- and age-matched in each experiment as much as possible.

### Tumor Cell Culture

The murine oropharyngeal cell line MOPC (C57BL/6-derived, HPV16 E6/E7^−^) was kindly provided by J. Lee (Sanford Research/University of South Dakota, Sioux Falls, SD, USA) and cultured as described previously (43). MOPC^EGFP^ cells were generated by lentiviral gene transfer using a pCL6IEGwo empty vector (44) as previously described (45). Cells were washed twice in phosphate buffer saline (PBS) before injection into mouse dermis under sterile conditions.

### Tumor Model

Syngeneic murine HNSCC line MOPC^EGFP^ were injected superficially in the dermis of the outer dorsal ear for intravital microscopy. Approximately 3 min before tumor cell injection the ear was depilated using commercially available depilatory cream, applied <2 min. Under Ketamin/Xylazin (100/20 mg/kg body weight) anesthesia ~10 μl of 20^*^10^6^/ml cell suspension in PBS was injected into the ear dermis using a 30-gauge cannula.

### Contralateral Day 3 Tumor

In one experiment, on day 3 after tumor cell inoculation, AZD5069 and vehicle-treated Catchup^IVM−red^ mice were injected with a second tumor into the contralateral outer ear dermis. Tumor injection and CXCR2 blockade was performed as described below.

### CXCR2 Antagonism

CXCR2 was blocked with small molecule antagonist AZD5069, which was provided by AstraZeneca. AZD5069 was diluted in vehicle solution consisting of 1.14% w/w HP-β-cyclodextrin and 0.5% Hydroxy propyl methyl cellulose (HPMC 6 cps) in 0.1 mM Carbonate buffer (pH 9.5–10). One-hundred microliter of 10 mM AZD5069 or vehicle solution only (for control group) was continuously administered twice daily (12 h interval) via oral gavage starting 12 h before tumor cell injection.

### Intravital 2-Photon Microscopy

Imaging of MOPC^EGFP^ tumors in the outer dermis of the dorsal ear was performed non-invasively on costume build water heated aluminum stage. The ear was gently mounted on pre-warmed aluminum block using Vaseline, covered with a cover slip and encumbered with a 1 cm diameter metal ring. PBS was used as immersion medium beneath and above cover slip sealed with Vaseline to prevent drain. Long term anesthesia was controlled via intubation narcosis and mechanical ventilation (1.5% Isoflurane in O_2_) using capnography to maintain physiologic ventilation (expiratory CO_2_: ~20 mmHg). Mice were injected with 10 μL of a 1 mM QTracker® 655 (Life Technologies, Darmstadt, Germany) solution i.v. to visualize blood vessels before constant observation in a Leica TCS SP8 MP microscope (Leica Microsystems, Mannheim, Germany) with simultaneous detection via hybrid-reflected light detectors and photomultiplier tubes with a HCX IRAPO L253/0.95 water objective. If not otherwise indicated excitation was performed at 960 nm using a Coherent Chameleon Vision II Ti:Saph-Laser (Coherent LaserSystems, Göttingen, Germany). The following filter settings were used: collagen (second harmonic generation, SHG) BP485/30; neutrophils (tdTomato transgene) BP585/50, blood vessels (QTracker®) BP660/30, tumor cells (EGFP) BP525/50. Raw data were reconstructed with Imaris (Bitplane, Zurich, Switzerland) for quantitative analysis and generation of representative pictures and videos.

### Statistical Analysis

Data were analyzed using GraphPad Prism Software (GraphPad Software, Inc., La Jolla, CA, USA). Statistical significance was assessed with paired or unpaired two-tailed *t*-test for the comparison of two groups and two-way ANOVA with Bonferroni post-tests for the comparison of multiple groups if not otherwise indicated. Results were considered statistically significant at *p* ≤ 0.05.

## Results

### Establishment of a Longitudinal Intravital Imaging System to Monitor TAN Mobility and Migration During Early Engraftment of Tumor Cells

**Figure 1:**
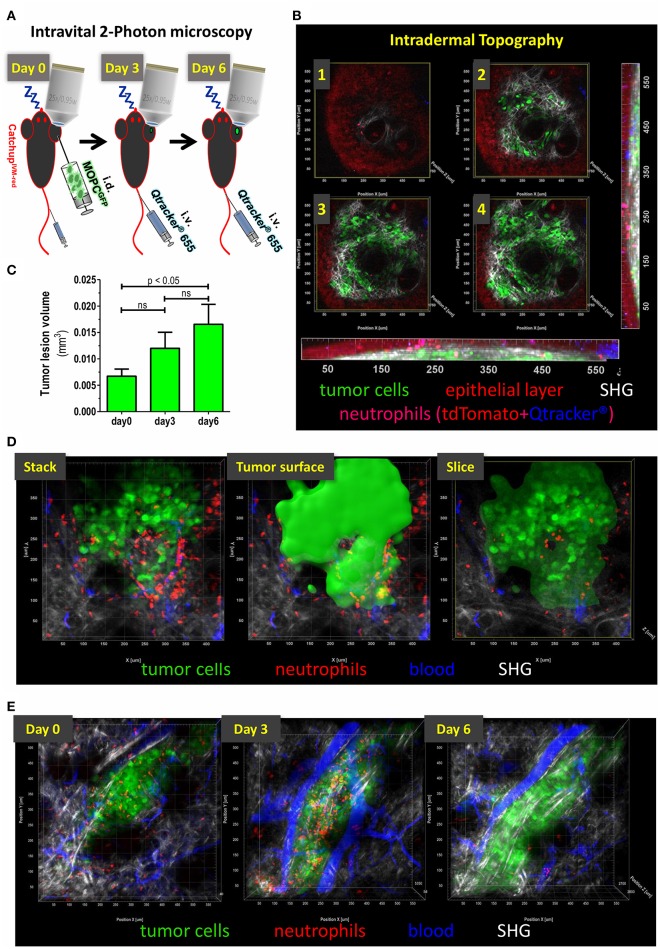
Topography of small intradermal lesions of tumor cells. **(A)** Experimental scheme for longitudinal analysis of spatio-temporal dynamics of TAN. After narcosis Catchup^IVM−red^ mice were intradermally injected with MOPC^EGFP^ cells into the dorsal ear skin (day 0). Directly before imaging Qtracker® vascular label 655 was injected i.v. into the tail vein or retrobulbar to visualize blood vessels. Imaging procedure on the same tumor lesion was repeated on days 3 and 6. **(B)** Images 1–4 show single cross sections in different depth in a multidimensional 2-Photon stack of an advanced tumor (>day 10). Orthogonal maximum intension projection (MIP) in the x-z-plane (bottom) and y-z-plane (right). Epidermal layer shows a weak autofluorescent signal in the tdTomato emission spectrum (red). Neutrophils show highly fluorescent tdTomato signals and weakly accumulate Qtracker® over time (here 1.5 h post-injection is shown). After fluorescence overlay, neutrophils are displayed in magenta. This enables additional differentiation from autofluorescence by mixed color. Only residual Qtracker® (blue) from leakage or clotting and no blood staining is visible since *i.v*. injection occurred 1.5 h prior to image acquisition in this case. MOPC^EGFP^ tumor cells are in green. Collagen fibers of the basal membrane and dermal matrix are visualized through SHG signal (white). Image 1 represents the epidermal-basal membrane border. **(C)** Means (+/– SEM) of tumor lesion volumes at different time points after tumor cell injection. Day 0 = 120–180 min after tumor cell injection. *N* = 6 animals. **(D)** Definition of tumoral compartments. Tumor volume was assessed by semi-automated surface generation of tumor cells (solid green). TAN inside tumor surface area were termed “intratumoral,” cells outside were designated as “peritumoral.” Cross-section through tumor volume reveals intra- vs. peritumoral TAN. **(E)** Intravital multidimensional 2-Photon images of representative tumor cell lesions in MIP from days 0 (120 min after tumor cell injection), 3, and 6 are depicted. 3D reconstruction was performed with Imaris® (Bitplane).

At first, we established technical requirements crucial for high quality, unperturbed, longitudinal imaging of TAN in early tumor cell lesions. Maintaining body temperature is important for preserving normal physiology of mice during prolonged and longitudinal imaging. Common heating pads are unsuitable for this purpose, since periodical heating leads to relevant material expansion and contraction with enormous shifts in z-direction. To circumvent this problem, we designed a water heated aluminum stage with an external heating unit, which was constantly perfused with 36°C warm water. After narcosis, depilation of the ear, tumor cell injection and *i.v*. blood labeling the mouse ear was gently mounted with petroleum jelly (Vaseline®) in prone position on pre-warmed aluminum block and covered with a glass cover slip. For stable long-term imaging conditions (>1 h) endotracheal intubation and controlled isoflurane narcosis (1.5% Isoflurane in 100% O_2_) with constant capnometry (FetCO_2_ = 2–3%) proved successful. Continuous capnometry ensured appropriate ventilation, sufficient narcosis depth, tolerability and an adjusted recovery phase. For short-term imaging periods (<1 h) *i.p*. ketamine narcosis without endotracheal intubation was sufficient. Using these procedures intravital imaging was performed on days 0, 3, and 6 (Figure 1A).

To this end, following the adoptive transfer of ~150,000 cells of the HNC cell line MOPC^EGFP^ (45), an appropriate superficial tumor cell lesion was identified with epifluorescence and navigation through oculars. The autofluorescence of epidermal cells followed by overlay with the second harmonic generation (SHG) signal of the basal membrane during multiphoton acquisition permitted navigation through skin layers (Figure 1B). Mean size of the lesion analyzed inside the field of view increased over time from ~0.007 mm^3^ (day 0, 120–180 min after injection) to 0.017 mm^3^ (day 6) (Figure 1C). Within the tumor cell lesion, we identified TAN in two distinct regions relative to the tumor cell mass. The center of a compact tumor lesion, consisting of densely packed tumor cells, was considered intratumoral and TAN localized in this area were designated intra-TAN. The directly adjacent, SHG signal/collagen rich, area within the field of view was termed peritumoral compartment. The peritumoral compartment was defined as a maximum distance of 250 μm from the tumor margin, which was expected to be in reach of paracrine tumoral conditioning factors, but without direct tumor cell contact (Figure 1D; Supplementary Video 1). TANs in this region were termed peri-TAN. Using our model, we could routinely record longitudinal sessions of TAN imaging in single tumor lesions from day 0 (up to 3 h post tumor cell injection) until days 3 and 6 post injection (Figure 1E).

This experimental model therefore has provided a reliable method for longitudinal monitoring of unmanipulated TAN in small newly established tumor cell lesions with high resolution and in the context of two different spatial compartments of the tumor microenvironment.

### Dynamics of Early Neutrophil Infiltration Into the Tumor Lesion

**Figure 2:**
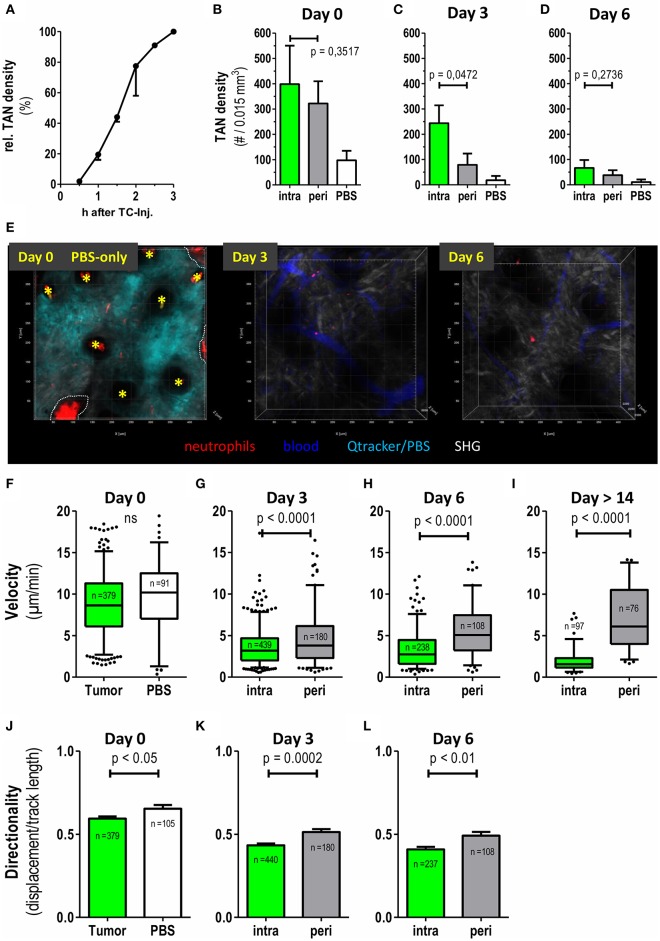
Differential recruitment and migratory patterns of intratumoral vs. peritumoral neutrophils. Catchup^IVM−red^ mice were injected with MOPC^EGFP^ cells into the dorsal ear dermis. TAN infiltration and motility was assessed by transdermal intravital 2PM. **(A)** Time course of TAN recruitment during the first 3 h after tumor cell inoculation. TAN density at 3 h was defined as 100% (mean with SEM of 2 representative mice is shown). **(B–D)** Neutrophil densities were quantified in intratumoral (green bars) and peritumoral (gray bars) compartment and after PBS injection only (white bars) at days 0, 3, and 6 and depicted as number of TAN per 0.015 mm^3^ tumor tissue volume (*n* = 6 mice for tumor and *n* = 2 mice for PBS). **(E)** Representative 2-D still images generated from three-dimensional multiphoton images in maximum intension projection (MIP) demonstrating low neutrophil recruitment and persistence in control Catchup^IVM−red^ mice injected with PBS+Qtracker® at day0. At day 0 the Qtracker dye (+PBS) (cyan) was injected into the ear dermis only. On day 3 and 6 blood vessels were labeled by i.v. Qtracker® (blue) injection. Yellow asterisks mark hair follicles (day 0). Dotted white line indicate areas of invaginated epidermal layer (wrinkle) with high red autofluorescence. Scattered neutrophils are represented by cell-associated red signals in the tissue parenchyma. **(F–I)** Velocities of neutrophils in the tumor compartments assessed by semi-automated tracking. **(J–L)** Migration of TAN in **(F–H)** was also analyzed for directionality (track length/displacement). **(F–L)** *n* = 4 for tumor bearing mice, *n* = 2 for PBS, *n* = 3 in **(I)** and cumulative number of analyzed single neutrophils depicted in each plot. Statistical significance was assessed with paired *t*-test **(B–D)** and unpaired two-tailed *t*-test **(F–L)**, α = 0.05. Mean +/– SEM is shown in bar graphs and 5–95% percentiles in boxplots. 3D reconstruction, quantification and tracking were performed with Imaris® (Bitplane).

Due to their small size, very early tumor lesions are not readily accessible to classical histological preparation and analysis. Hence, intravital 2PM was especially suited to monitor immune cell dynamics in these very early tumor cell lesions. Supplementary Video 2 records TAN infiltration between 45 and 120 min after tumor cell injection. At 60 min post injection, high numbers of highly migratory neutrophils started to infiltrate the tumor lesion (Supplementary Video 2). This influx followed sigmoid kinetics over the first 3 h (Figure 2A) and at 3 h post injection substantial numbers of neutrophils infiltrated the tumor injection site. In order to test whether the injection procedure itself may cause accumulation and recruitment of neutrophils we monitored injections of PBS (Figure 2E). While PBS injection indeed stimulated the recruitment of a smaller number of neutrophils, this influx was clearly low-level compared to the tumor cell-induced recruitment (Figures 2B,E). In addition, neutrophils, induced by this initial mechanical stimulus, showed short persistence and almost completely disappeared from the injection site by days 3 and 6 (Figures 2C–E).

We next investigated numbers and migration of individual TAN. While intra-TAN were mostly in contact with the carcinoma cells themselves, peri-TAN were in contact with the surrounding normal or stromal tissue and the extracellular matrix. At 2–3 h post injection, a substantial number of neutrophils was present in both compartments. At day 3 the frequency of peri-TAN was already strongly decreased from 322 cells/0.015 mm^3^ (day 0) to 79 cells/0.015 mm^3^, with further reduction by day 6 (Figures 2B–D). In contrast, intra-TAN frequencies remained at high levels until day 3 and only decreased to lower levels by day 6 (Figures 1B, 2B–D).

We next quantified and compared the motility of TAN in these two compartments. At day 0, both intra-TAN and peri-TAN were highly migratory displaying an average velocity of 8.8 μm/min. This velocity was comparable to neutrophils recruited in response to PBS injection, suggesting that tumor cells strongly increased recruitment of neutrophils over the injection trigger as such (Figure 2B), but did not further modulate their speed (Figure 2F). We did not compare migratory properties of intra- vs. peri-TAN at day 0, since neutrophils rapidly interchanged between compartments at this early point in time, making a clear allocation impossible. Instead the comparison of velocity of intra-TAN and peri-TAN was performed starting at day 3 and then followed up for at least 10 additional days. We observed that the velocities of intra-TAN strongly decreased during tumor development and TAN in larger developed tumors (day 14 or later) displayed a rather sessile phenotype (Figures 2G–I and Supplementary Video 3). Interestingly, and in contrast to directly tumor cell-associated neutrophils, peri-TAN increased their velocity over time. By day 14, this resulted in a substantial difference in velocity of intra- vs. peritumoral TAN (Figure 2I). Next, we analyzed the directionality of TAN migration over time. At day 0 (1 to 3 h after injection) the infiltration of neutrophils into the lesion was directional (Figure 2J, directionality > 0.5). In contrast, at days 3 and 6, the overall directionality of TAN decreased over time, with peritumoral TAN constantly displaying a slightly higher directionality than intratumoral TAN (Figures 2K,L). Supplementary Video 4 supports this finding.

In conjunction, these data establish previously unknown time-dependent differences in recruitment, persistence and migratory behavior of TAN located in either the intratumoral or the peritumoral area of the tumor microenvironment.

### Effect of CXCR2 Blockade on TAN Recruitment Into Tumors

**Figure 3:**
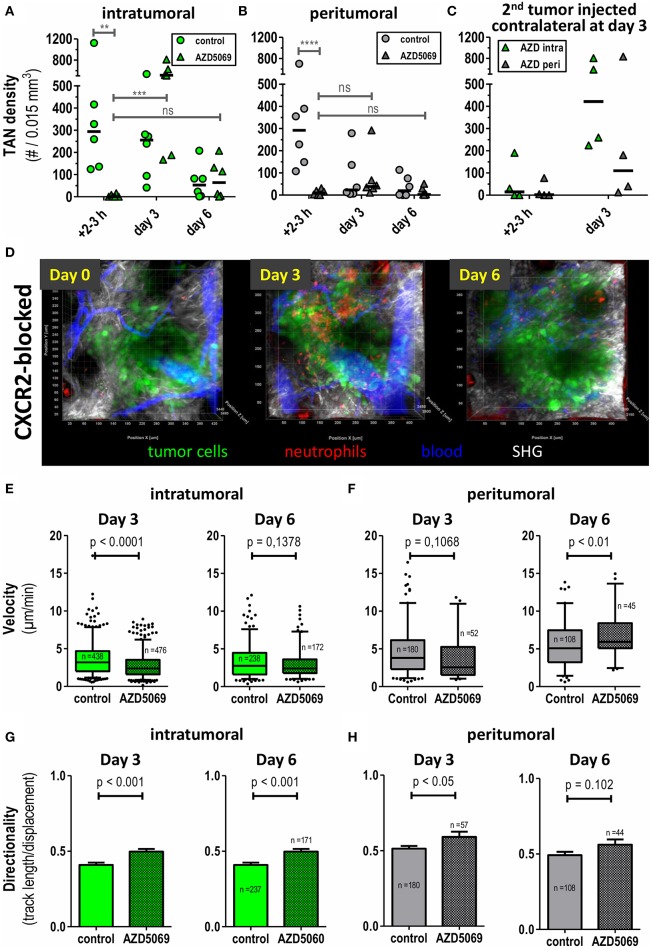
Intratumoral and peritumoral TAN are differentially affected by CXCR2 blockade to AZD5069 treatment. Catchup^IVM−red^ mice were injected with MOPC^EGFP^ cells into the dorsal ear dermis. TAN infiltration and motility was assessed by transdermal intravital 2PM. To block CXCR2 AZD5069 was orally administered twice daily starting from day−1 before tumor cell inoculation until the end of the experiment. Intratumoral **(A)** and peritumoral **(B)** TAN infiltration was quantified as neutrophils per 0.015 mm^3^. Note the efficient reduction of both intratumoral and peritumoral TAN by AZD5069 at day 0. Also note the rebound of intratumoral, but not peritumoral, TAN under AZD5069 treatment at days 3 and 6. **(C)** Three days after injection of the primary tumor, AZD5069-treated mice received a second tumor injection into the contralateral ear dermis. TAN density was determined in intra- and peritumoral areas 2–3 h and 3 days after 2nd tumor injection. **(D)** Intravital multidimensional 2-Photon images of representative tumors of AZD5069 treated mice in maximum intension projection (MIP) on days 0, 3, and 6. Velocities and directionality of intratumoral **(E,G)** and peritumoral **(F,H)** TAN in AZD5069-treated compared to control mice were determined on days 3 and 6 by intravital 2PM and semi-automated tracking (pooled data from *n* = 4 mice per group). Statistical significance of difference was assessed with unpaired two-tailed *t*-test (α = 0.05). Data in **(A–C)** are individual mice and the bar represents the median. Data in **(E–F)** are presented as box-plots with whiskers indicating the 5–95% percentile. In **(G–H)** barplots of means with SEM. In **(A–C)** each symbol represents one mouse. ^**^p < 0.01, ^***^p < 0.001, ^****^p < 0.0001, ns = *p* > 0.05. 3D representation, quantification, and tracking were performed with Imaris® (Bitplane).

We have previously shown that TAN in this MOPC tumor model express high amounts of CXCR2 on their surface (45). Expression of CXCR2 ligands in the tumor microenvironment is believed to be a major pathway of TAN recruitment in murine tumor models (20, 46–48) and even in human HNC patients (3). Given the tumor-promoting role of TAN, interference with CXCR2/CXCR2-ligand interaction, has been proposed as a means to limit the pro-tumorigenic activity of TAN. Against this background, we investigated how CXCR2 blockade would affect frequencies and migratory patterns of TAN in this model. Consistent with this idea the small molecule CXCR2 antagonist, AZD5069 effectively blocked the influx of TAN into both the intratumoral and peritumoral areas at early time points after tumor cell inoculation (left columns, 2–3h, Figures 3A,B). However, unexpectedly, intratumoral TAN rebounded by days 3 and 6 despite AZD5069 treatment. Thus, CXCR2 blockade was unable to limit the recruitment of intra-TAN to intratumoral areas at days 3 and 6 (Figure 3A, compare Figure 3D for the respective still images of videos). This inability was in contrast to the durable inhibitory effect of AZD5069 on the frequency of peri-TAN, which did not show a significant rebound (Figure 3B). In fact, in most experiments CXCR2 blockade still maintained peri-TAN density to levels below 70 cells/0.015 mm^3^ on days 3 and 6 (Figure 3B) while intra-TAN reached levels comparable to or even higher than control mice by day 3 and later (Figure 3A). This rebound of intra-TAN occurred despite reduced levels of circulating neutrophils in AZD5069-treated mice until day 6 (Supplementary Figure 1). To confirm that AZD5069 was still generally active at day 3, we injected a second tumor at the contralateral side at this time point (Figure 3C). In this tumor, CXCR2 blockade still effectively inhibited the immediate recruitment of TAN into the tumor lesion at 2–3 h post injection. This indicates the *in vivo* activity of the compound despite the inability to exclude intra-TAN from tumors injected 3 days earlier in the same animal. Strikingly, also in the secondary tumor, AZD5069 lost effects on intra-TAN 3 days post injection (Figure 3C) suggesting that this effect is mediated by a change in tumor biology rather than TAN biology. Consistent with observations in primary tumors, in most experiments CXCR2 blockade retained its inhibitory activity on the recruitment of peri-TAN by 3 days post injection also for the 2nd challenge tumor.

Having analyzed the effects of CXCR2 blockade on TAN densities, we then assessed the effect of CXCR2 blockade on fundamental motility parameters of TAN in both compartments. In day 0 intravital imaging showed the trafficking of neutrophils through vasculature directly adjacent to tumor cell lesions. As expected, also signs of neutrophil adhesion and rolling could be observed. Importantly, and confirming data from Figures 3A,B, the extravasation of TAN from vessels into the tumor lesion was completely abrogated by CXCR2 blockade at this time point (Supplementary Video 5). Since AZD5069 treatment completely prevented TAN recruitment to tumor lesions at the day of injection, motility was subsequently only analyzed at days 3 and 6 post injection. Interestingly, and despite its inability to reduce the recruitment of TAN to the intratumoral lesion at this time point (Figure 3A), AZD5069 still significantly reduced the motility of intra-TAN at day 3 (Figure 3E). However, at day 6 this effect of CXCR2 blockade on intra-TAN over vehicle treated animals was lost. A similar pattern of response was found for AZD5069 effects on the small numbers of peri-TAN. As depicted in Figure 3F, AZD5069 reduced the velocity of peri-TAN only at day 3 and not at day 6. Instead, the small number of peri-TAN, which infiltrated the tumors in the presence of CXCR2 blockade at day 6, seemed to display substantial mobility with a mean velocity above 5 μm/min (Figure 3F). In summary, the effects of CXCR2 blockade on the migration demonstrated clear differences between intra- and peri-TAN. In contrast, we observed a coherent effect of CXCR2 blockade on the directionality of TAN migration in both compartments. Here, AZD5069 treatment equally increased the directionality of TAN migration in both compartments (Figures 3G,H).

These data show, that, in addition to its distinct inhibitory effects on intra- and peri-TAN recruitment, AZD5069 also affects the intratumoral motility and directionality of these TAN subtypes in small tumor lesions.

## Discussion

In this study, we demonstrate the establishment of an experimental system of unperturbed longitudinal tumor-associated neutrophil (TAN) observation in the living mouse. To this end, we used a tumor cell injection model in the murine ear dermis. While this model has many advantages in terms of the imaging technology, it also has apparent limitations. Notably, injection models, particularly with respect to early growth phases do not fully recapitulate the complex multi-component tumor-stroma available in selected chemically induced or transgenic models. Despite these limitations, syngeneic transplantation models are very frequently used for experimental research and important aspects of immuno-oncology are being investigated in such models (45, 47, 49, 50). In terms of *in vivo* imaging, many experimental models to date require major surgical intervention to make tumor lesions accessible to imaging technologies (51, 52). This constitutes a trauma with subsequent effects on immune cell infiltration and behavior. Our model utilizes a minimally invasive procedure allowing for longitudinal long-term observations of the invasion of unperturbed TAN into a growing tumor. Although artificial disruption of tissue integrity occurs during intradermal injection in this model, the degree of damage is comparable to human tumor-associated wounds and inflammation, which are actually induced by invasive malignant progression or iatrogenic biopsies and surgery (53, 54). Our model, therefore, recapitulates certain aspects of regular tumor development in patients with cancer. In addition, we investigated the stimulus by PBS injection itself. By comparing neutrophil dynamics in PBS-only lesions with tumor cell injection, we could show that tumor cells are the major source of neutrophil attraction and exclusively induce persistence in this model. Tumor lesions showed four times greater neutrophil densities than PBS lesions. Further, the neutrophil influx in PBS lesions was transient; and resolved to background by day 3, while TAN recruitment was durable over >6 days of observation.

Interestingly, we observed the formation of densely packed areas of tumor cells within 3 h after tumor cell injection and tumor cells showed tight microscopic cell-contacts. It is tempting to speculate that the injection of tumor cells and the formation of dense tumor cell areas also influences the biology of the surrounding stromal tissue. Our intravital imaging shows effective triggering of TAN recruitment into what we designated “intratumoral” and “peritumoral” (surrounding stromal) areas. Thus, at these early time points, most likely both tumor cell-derived and stromal cell-derived factors trigger TAN recruitment (22, 23, 55). At later time points, intra-TAN showed prolonged persistence and reduced motility, consistent with *in vitro* observations demonstrating recruitment and delayed apoptosis of neutrophils in response to tumor-derived factors (12, 56).

Most tumors consist of tumor cell islands and surrounding parenchyma or non-malignant stroma cells. Recently, we demonstrated a differential prognostic role of stromal vs. tumoral inflammation in head and neck squamous cell cancer (HNSCC) patients (57). Considering the emerging prognostic relevance of tumor-stroma constitution and sublocalization of immune infiltrates in solid tumors (58, 59), we especially focused on separate analyses of tumoral compartments in this study and could indeed find striking differences in TAN frequencies, motility and CXCR2-dependent regulation with regard to TAN localization.

A key finding was the reduced directionality and velocity of intra-TAN compared with peri-TAN. This reduced migratory activity of intra-TAN could be a possible reason for the persistence of high TAN densities in the intratumoral compartment beyond day 3 as opposed to the rapid decrease of peri-TAN frequencies. Additional evidence for the intratumoral persistence of intra-TAN comes from an analysis of adoptively transferred peripheral blood leukocytes from Catchup^IVM−red^ mice together with tumor cells into the ear dermis of C57BL/6 mice (Supplementary Figure 2). Here, we observed viable migrating adoptively transferred TAN until day 3 after transfer, suggesting that at least a certain number of intra-TAN can persist for up to 3 days in the tumor lesion. In contrast, the increased mobility of peri-TAN may lead to an increased chance of contact to distracting cues from sites away from the tumor lesion. It remains to be shown, which mechanisms are active in recruiting peri-TAN away from the tumor. Also delayed apoptosis of intra- vs. peri-TAN is a potential mechanism that might explain our findings (56, 60). New models utilizing photoactivatable GFP-transgenic neutrophils have recently been published (61) and could be used to further decipher the fate and function of intra-TAN after recruitment into the tumor core area.

CXCR2 is a major signaling pathway in neutrophil recruitment in tumors and non-malignant neutrophil-driven inflammatory diseases (34, 62–64). In a previous study, using the same MOPC cell line, we have demonstrated expression of KC and MIF by MOPC tumors (45). Interestingly, in the present study, we observed differential effects of CXCR2 blockade on TAN localized in either the intratumoral or peritumoral tissue areas. The fact that CXCR2 blockade efficiently blocked primary TAN influx on day 0 suggests a major role of the CXCR2 pathway in driving acute TAN recruitment. This is consistent with published intravital data in zebrafish larvae where neutrophil recruitment to wounds is abrogated by CXCR2 antagonists (65). However, in addition tumors may develop CXCR2 independent mechanisms of TAN recruitment which lead to stable TAN infiltration beyond day 3. Alternatively, TAN residing in specialized niches of tumors might produce factors that recruit additional TANs, as has been shown recently for HGF, that is produced by TAN and recruits additional TANs via c-Met signaling (66). Similar observations have been made for neutrophils in necrotic lesions that induce a feed-forward loop for the recruitment of other neutrophils via leukotriene B_4_ (67). Future work needs to address, which mechanism is active in our model. Interestingly, in a model of zebrafish wounding additionally to the initial recruitment of neutrophils also the resolution of inflammation seemed to depend on CXCR2 (65).

The chemokine receptor CXCR2 is primarily expressed on mature neutrophils. However, in tumor hosts often an expansion of immature neutrophils occurs (45). Immature circulating neutrophils normally express low or no CXCR2. Evrard et al. recently showed that tumor bearing mice display elevated numbers of immature CD101^−^ neutrophils in blood and pancreatic tumors compared to naive mice (68). Those cells showed low surface expression of CXCR2. Interestingly, in this model CXCR2^−^ immature cells were still capable of normal tissue infiltration and interstitial migration. Hence, antagonizing CXCR2 using AZD5069 may selectively allow CXCR2^−^ immature neutrophils only to infiltrate transplanted tumors in our model. By contrast, CD101^−^ immature neutrophils only account for 1 to 5 % isolated neutrophils in blood and 5 to 16% of isolated neutrophils from pancreas in animals with low or high tumor burden, respectively (68). However, in our tumor model the numbers of intra-TAN in CXCR2-blocked Catchup^IVM−red^ mice even exceeded those of vehicle treated animals on day 3. In addition, using the same tumor cell line model as in this study, we recently reported that in both, naïve and tumor-bearing C57BL/6 mice, neutrophils in the blood, bone marrow and spleen consistently express considerable amounts of CXCR2 (45). The high TAN content under CXCR2 blockade occurs despite reduced systemic levels of circulating neutrophils (Supplementary Figure 1). Next to TAN-intrinsic mechanisms, it might still be possible, that the tumor changes its phenotype in a co-evolution with TAN or other infiltrating immune cells, which should be investigated in future studies. Furthermore, tumor cell triggered differential chemokine modifications in the densely packed intratumoral compartment may differ from chemokine processing and constitution in the peritumoral compartment. For neutrophil activating chemokines, it is known that their effect is modulated by post-translational changes like nitration or binding to glycosaminoglycans (69). Additionally, differential constitution in terms of extracellular matrix or extracellular proteolytic activity of both compartments may lead to differential conformational changes of the present chemokines to predominantly monomers or dimers. Since it was shown for CXCL1 that monomeric and dimeric form display differential activity in CXCR2 binding leading to a possible fine-tuning of chemokine-receptor pair effects (70), it is plausible that differential chemokine constitution in both compartments may lead to differential migratory properties of TAN.

In summary, our data represent the first targeted observation of unperturbed TAN in the living mouse during very early tumor establishment *in vivo*. Despite known and obvious limitations in terms of tumor cell development, our transplantable tumor model for intravital imaging reflects certain elements of human tumor cell biology and allows longitudinal tracking of spatio-temporal dynamics of unperturbed genetically labeled TAN. Clearly, our data suggest that infiltration of intratumoral lesion and peritumoral stroma are differentially regulated in terms of chemo-attractive and repulsive cues including the respective chemokine receptors and ligands involved. Interestingly, CXCR2 antagonism by AZD5069 is ineffective in preventing Ly6G^+^ cell recruitment to tumor lesions at latter timepoints. Additional studies are required to decipher the complex bi-directional cross-talk of tumor tissue and TAN responsible for this dynamic interplay. Preliminary data in our group indicate strong tumor promoting features of intratumoral TAN (data not shown). Hence, our intravital system opens the possibility to further functionally characterize these distinct classes of TAN in different tumoral compartments and thereby enables unpreceded insight into TAN biology in the living animal.

## Ethics Statement

This study was carried out in accordance with the recommendations of the animal ethics committee of the state of North Rhine–Westphalia, Germany, and German guidelines for experimental animal welfare. The protocol was approved by the animal ethics committee of the state of North Rhine–Westphalia, Germany.

## Author Contributions

SB, SS, and MG: conceptualization. SS and AGr: investigation. SS, SB, MG, and MU: writing manuscript. SB, MG, and SS: data curation and analysis. SB, MU, MG, AGr, AGö, and BG: resource. SB, MG, MU, and AGö: reviewing manuscript.

### Conflict of Interest Statement

AGö is a consultant for and has equity interest in Evox Therapeutics Ltd., Oxford, UK. MU is an employee of AstraZeneca and holds shares in AstraZeneca. The remaining authors declare that the research was conducted in the absence of any commercial or financial relationships that could be construed as a potential conflict of interest.

## Abbreviations

2PM: two-photon microscopy
a.dest: distillated water
ADCC: antibody-dependent cellular cytotoxicity
BP: bandpass
COPD: chronic obstructive pulmonary disease
ECM: extracellular matrix
FetCO_2_: end-tidal CO_2_-fraction
G-MDSC: granulocytic myeloid-derived suppressor cells
HNC: head and neck cancer
HNSCC: head and neck squamous cell carcinoma
i.d.: intradermal
i.p.: intraperitoneal
i.v.: intravenous
LP: longpass
MIP: maximum intension projection
NIR: near infrared
PBS: Phosphate buffered saline
SHG: second harmonic generation
TAN: Tumor-associated neutrophils
TME: Tumor microenvironment.
