# Supplementary material for: Distinct Spatio-Temporal Dynamics of Tumor-Associated Neutrophils in Small Tumor Lesions
Source: Front Immunol. 2019 Jun 25;10:1419. doi: 10.3389/fimmu.2019.01419 (PMC6603174; doi:10.3389/fimmu.2019.01419)
Supplement: Supplementary file 6 [file Data_Sheet_1.pdf]

Supplementary Material

for S. Sody et al.:

Distinct spatio-temporal dynamics of tumor-associated neutrophils in small tumor lesions

1 MATERIALS AND METHODS

1.1 Peripheral blood neutrophil count determination

C57BL/6 mice were treated with AZD5069 in vehicle solution or vehicle solution only by orally gavage twice daily. Immediately before next gavage, a peripheral blood droplet was obtained via tail vein puncture. Neutrophil fraction of all leucocytes was analyzed in whole blood droplet staining of neutrophils was performed with α-Ly-6G-PerCP/Cy5.5 1A8 (BioLegend, Fell, Germany), after erythrocyte lysis in aqua dest for 20 sec. and flow cytometry. Leucocytes were gated on FFS/SSC scatter properties and the percentage of Ly-6G-positive cells was determined.

1.2 Catchup$^{IVM\text{-}red}$-PB-leucocyte tumor co-injection

Approximately 500 µl peripheral blood of a single Catchup$^{IVM\text{-}red}$ mouse was collected through heart puncture. After centrifugation erythrocyte lysis was performed twice with aqua dest for 20 seconds. The pellet was resuspended in 50 µl PBS solution containing $1{*}10^6$/ml MOPC^{EGFP} cells. Under Ketamin/Xylazin (100/20 mg/kg body weight) anesthesia, 10 µl MOPC/PB-leucocyte cell suspension was injected into the ear dermis of a C57BL/6 mouse using a 30-gauge cannula after local depilation as described before and intravital two-photon microscopy was performed.

2 Supplementary Videos

Video 1. Definition of tumoral compartments. Two-photon microscopy of a day 6 tumor through intact epidermis of the outer dorsal ear of an AZD5049-treated Catchup$^{IVM\text{-}red}$ mouse. Video showing maximum-intensity projection of a three-dimensional stack with neutrophils (red), tumor cells (green), SHG (white) and Qtracker® (blue). Semi-automated surface generation for intratumoral compartment segmentation in solid green. Intratumoral TAN are marked turquoise, peritumoral TAN yellow. Representation was performed using Imaris® (Bitplane).

Video 2. Early recruitment and infiltration of neutrophils into a tumor lesion. Two-photon microscopy was performed through intact, previously depilated epidermis of the outer dorsal ear of Catchup$^{IVM\text{-}red}$ mice. Time-lapse image sequence displayed in maximum-intensity projection of a three-dimensional stack indicated by frame (µm), showing neutrophils (red), tumor cells (green), SHG (white) and Qtracker® (blue). Video starts 45 min after tumor cell injection. Elapsed time since acquisition is displayed as hours:minutes:seconds. Representation was performed using Imaris® (Bitplane).

Video 3. Sessile intratumoral TAN in advanced tumor. Two-photon microscopy time-lapse imaging of a day 20 tumor through intact epidermis of the outer dorsal ear of a Catchup$^{IVM\text{-}red}$ mouse. Maximum-intensity projection of a three-dimensional sequence showing tumor cells (green), SHG (white) and neutrophils (red) with tracks (rainbow colored tails). Elapsed time since acquisition is displayed as hours:minutes:seconds. Representation was performed using Imaris® (Bitplane).

Video 4. Differential migratory pattern of TAN in the intratumoral and peritumoral compartment. Day 6 tumor of an AZD5069 treated Catchup$^{IVM\text{-}red}$ mice imaged with two-photon microscopy through the intact, previously depilated epidermis of the outer dorsal ear. Time-lapse image sequence displayed in maximum-intensity projection of a three-dimensional stack indicated by frame (µm), showing neutrophils (red) and tracks (rainbow colored tails), tumor cells (green), SHG (white) and Qtracker® (blue). Elapsed time is displayed as hours:minutes:seconds. Representation was performed using Imaris® (Bitplane).

Video 5. Intravascular rolling and adhesion of CXCR2-blocked neutrophils on day 0. Two-photon microscopy time-lapse imaging of a day 0 tumor through intact epidermis of the outer dorsal ear skin of an AZD5069 treated Catchup$^{IVM\text{-}red}$ mouse. Maximum-intensity projection of a three-dimensional sequence showing tumor cells (green), SHG (white), neutrophils (red) and Qtracker® (blue). Framerate is 3 frames per minute. Neutrophils inside bloodstream can only be resolved when adherent to endothelium and drastically reduced velocity. Elapsed time since acquisition is displayed as hours:minutes:seconds.. Representation was performed using Imaris® (Bitplane).

3 Supplementary Figures

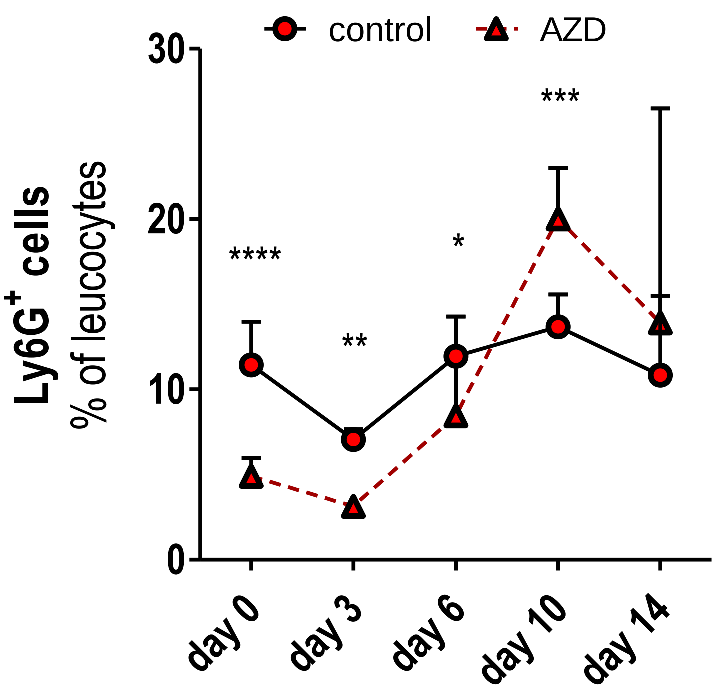

Supplementary Fig. 1. Peripheral blood neutrophil counts. Ear tumor bearing C57BL/6 mice were treated with AZD5069 in vehicle solution or vehicle solution only by oral gavage twice daily as described. Prior to gavage, a peripheral blood droplet was drawn to determine relative neutrophil frequencies in the peripheral blood on days 0, 3 and 6 (n=9 each), 10 (n=6) and 14 (n=3) with flow cytometry. Neutrophils were stained for Ly6G-positivity and relative fraction to whole leucocytes is depicted. Statistical analysis was performed with two-way ANOVA and Bonferroni post-tests. *p<0.05, **p<0.01, ***p<0.001, ****p<0.0001

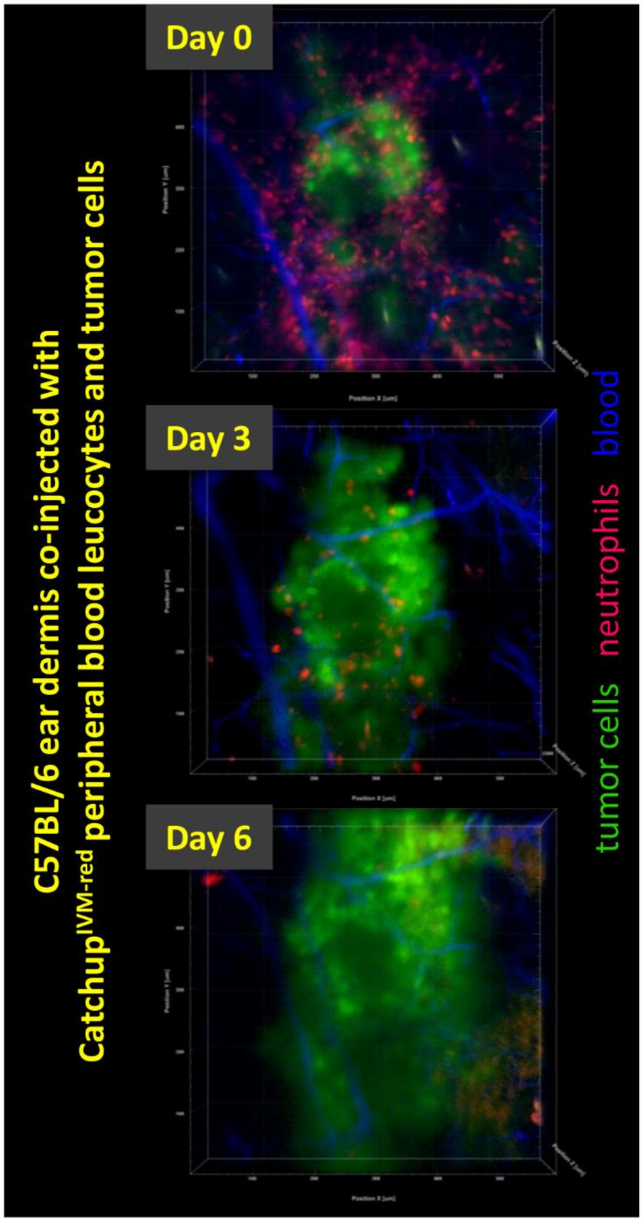

Supplementary Fig. 2. Persistence of co-injected Catchup$^{IVM\text{-}red}$-PB-leucocytes in a C57BL/6 mouse ear dermis. Intravital multidimensional confocal laser scanning fluorescent images of an early intradermal MOPC^{GFP} ear tumor co-injected with Catchup^{IVM}-PB-leucocytes in a C57BL/6-mouse at different time points as indicated (maximum intension projection). Until day 3 Catchup^{IVM}-neutrophils are detected suggesting a life time of at least 3 days. 3D representation was performed with Imaris® (Bitplane).
